# Geographic Analysis of Urologist Density and Prostate Cancer Mortality in the United States

**DOI:** 10.1371/journal.pone.0131578

**Published:** 2015-06-25

**Authors:** Nengliang Yao, Steven M. Foltz, Anobel Y. Odisho, David C. Wheeler

**Affiliations:** 1 Department of Healthcare Policy and Research, Virginia Commonwealth University, Richmond, VA, United States of America; 2 Department of Urology, Helen Diller Comprehensive Cancer Center, University of California, San Francisco, San Francisco, CA, United States of America; 3 Department of Biostatistics, Virginia Commonwealth University, Richmond, VA, United States of America; Stanford University School of Medicine, UNITED STATES

## Abstract

**Context:**

Financial and demographic pressures in US require an understanding of the most efficient distribution of physicians to maximize population-level health benefits. Prior work has assumed a constant negative relationship between physician supply and mortality outcomes throughout the US and has not addressed regional variation.

**Methods:**

In this ecological analysis, geographically weighted regression was used to identify spatially varying relationships between local urologist density and prostate cancer mortality at the county level. Data from 1,492 counties in 30 eastern and southern states from 2006–2010 were analyzed.

**Findings:**

The ordinary least squares (OLS) regression found that, on average, increasing urologist density by 1 urologist per 100,000 people resulted in an expected decrease in prostate cancer mortality of -0.499 deaths per 100,000 men (95% CI -0.709 to -0.289, p-value < 0.001), or a 1.5% decrease. Geographic weighted regression demonstrated that the addition of one urologist per 100,000 people in counties in the southern Mississippi River states of Arkansas, Mississippi, and Louisiana, as well as parts of Illinois, Indiana, and Wisconsin is associated with decrease of 0.411 to 0.916 in prostate cancer mortality per 100,000 men (1.6–3.6%). In contrast, the urologist density was not significantly associated with the prostate state mortality in the new England region.

**Conclusions:**

The strength of association between urologist density and prostate cancer mortality varied regionally. Those areas with the highest potential for effects could be targeted for increasing the supply of urologists, as it associated with the largest predicted improvement in prostate cancer mortality.

## Introduction

Uncertainty surrounding the implementation and impact of the Patient Protection and Affordable Care Act has further fueled the debate regarding the composition and distribution of the physician workforce. The graying of the American population, increasing prevalence of chronic disease, and improved access to care has led to concern regarding the adequacy of the physician workforce, both in primary and specialty care.[1–4] Financial and demographic pressures require an understanding of the most efficient distribution of physicians to maximize population-level health benefits.

When assessed at a national level, increased primary care physician density has been associated with improved cancer mortality rates.[5] Other studies have described a relationship between specialist physician supply and cancer mortalities.[6–10] The presence of a urologist in a county has been associated with lower urologic cancer mortality and the presence of colorectal and general surgeons has been associated with lower colorectal cancer mortality.[7,8] Researchers have also found lower melanoma mortality in areas served by dermatologists.[9,11] However, some work in other health care fields found conflicting and inconsistent results regarding the association of physician supply and cancer outcomes.[12,13] Those studies assume that the relationship between physician density and cancer outcomes is constant throughout the study area, and have not measured regional variation of the relationship between physician supply and cancer mortalities in statistical results.[7,8,14] Analyses of regression residuals often reveal that this assumption is not necessarily true (S1 and S2 Figs).[15,16] It is unlikely that one unit of increase in cancer specialist density in a high physician density region such as the Greater Boston area would have the same effect on cancer mortalities as in a low physician density region such as West Virginia.

In order to address these limitations of the existing literature and build on prior work,[7] we used geographically weighted regression (GWR) to study the relationship between physician density and cancer mortality rates. GWR models spatial variation in the relationship between the outcome variable and the explanatory variables.[15] Working at the county level, we sought to determine if increased urologist density was associated with lower prostate cancer mortality and if this relationship varied among counties in the United States.

## Study Data and Methods

### Data

County-level data measures of health resources were obtained from the Area Resource File (ARF) administered by the U.S. Health Resources Services Administration.[17] The ARF publishes the number of physicians by specialty per county, based on the American Medical Association Physician Masterfile. Physician (urologist, radiation oncologist, and primary care physician) density was defined as the number of physicians in each group per 100,000 residents in each county, weighted using data from 2006 and 2010. In addition, county health professional shortage area status and socio-economic indicators (the percentages of population over 65, non-white, and over 25 with a high school diploma, and per capita income) were abstracted from the ARF. The latitude and longitude of each county’s geographic centroid was obtained from the County and Equivalent Map (Census 2000) produced by the Geography Division of the U.S. Census Bureau.[18]

County-level prostate cancer mortality rates were obtained from the Surveillance, Epidemiology, and End Results (SEER) program of the National Cancer Institute (NCI).[19] Five-year aggregate mortality rates, which have been found to be reliable over time and space, were used.[20–23] The rates were age-adjusted by the direct method using five-year age groups, with the 2000 U.S. Census standard population as the reference. Data from counties with fewer than ten deaths are suppressed by the NCI (27% of eastern and southern counties). Counties with zero reported deaths were included. County-level prostate cancer incidence rates were obtained from the NCI State Cancer Profiles website.[24] Rates represent the average incidence from 2006–2010 and include men of all ages and ethnicities. Incidence rates were reported as the number of cases per 100,000 people and were age-adjusted to the 2000 U.S. standard population. To protect patient identities, data from counties with fewer than sixteen cases are suppressed by the NCI (4% of eastern and southern counties). Counties with zero reported cases are included. Since this is a county-level secondary data analysis, informed consent or institutional review board review was not required.

Western states were excluded from spatial analyses due to missing data. 56% of western counties were missing either mortality or incidence data, not including Alaska, Washington, Kansas, or Minnesota, which were completely missing mortality data. Of the 30 eastern and southern states included for analysis, data from 1492 out of 2069 (72%) counties were included. Data from the remaining 577 counties (many in western Texas) were missing because too few prostate cancer cases or deaths were reported. Data from Ohio and Virginia are not publicly available due to state regulations. S3 Fig visualizes the missing data issue in central states.

### Analysis

The spatial distribution of prostate cancer mortality rates across the study region was mapped to determine if geographic groupings (i.e., clusters) were evident. Spatial autocorrelation in the mortality rates was assessed using Moran’s I in the GeoDA software package with a weight matrix based on six nearest neighbors.[25] Permutation tests were used to indicate statistical significance of Moran’s I.

Regression analyses were performed in *R* (version 3.0.2) using the *GWmodel* package.[26] An ordinary least squares (OLS) regression model was created as a baseline comparator for GWR models. We included covariates as discussed in our prior work,[7] including urologists per 100,000 people, primary care MDs per 100,000 people, prostate cancer incidence per 100,000 men, hospital beds per 100,000 people, county classification as a Health Professionals Shortage Area (HPSA), metropolitan classification in the 2003 Rural-Urban Continuum Code scheme, the percentages of population over 65 years of age, non-white, over 25 years of age without a high school diploma, and per capita income. We added an indicator for a county having at least one radiation oncologist and treated primary care physician and urologist density as continuous variables.[7]

The spatial pattern of residuals in the OLS model has been examined and revealed that the relationship between physician density and cancer outcomes may not hold everywhere in the study area (S1 and S2 Figs).[15,16] We performed a GWR to obtain local coefficient estimates and adjusted approximate p-values based on the Benjamani-Yekutieli false discovery rate (FDR) method (see S1 File).[27,28] The range of GWR coefficient estimates was tabulated and urologist density coefficient estimates were mapped.

Another sensitivity analysis using health service area as the unit the analysis was planned but not executed, because most of the our data are only available at the county level and could not be easily aggregated into health service areas because of the missing data in counties with smaller population size. However, we expect the results would be essentially similar in the analysis of health service areas because GWR generally does not suffer from modifiable areal unit problem.[15]

### Collinearity diagnostics

Collinearity has been found to be especially problematic in GWR models and detailed collinearity diagnostics and model selection procedures are provided in the S1 File.[29] Diagnostic tools from the R package *gwrr* were used to explore problems with collinearity in the GWR models.[30] Based on the diagnostic tests, the variable for percentage of the population under 65 without health insurance was omitted to arrive at the final GWR model (see S1 File). In addition, given more than 90% of prostate cancer deaths are among men aged 65 or older and the median age of death is 80, most of prostate cancer survivors are Medicare beneficiaries.[31]

## Study Results

### Descriptive statistics

**Fig 1:**
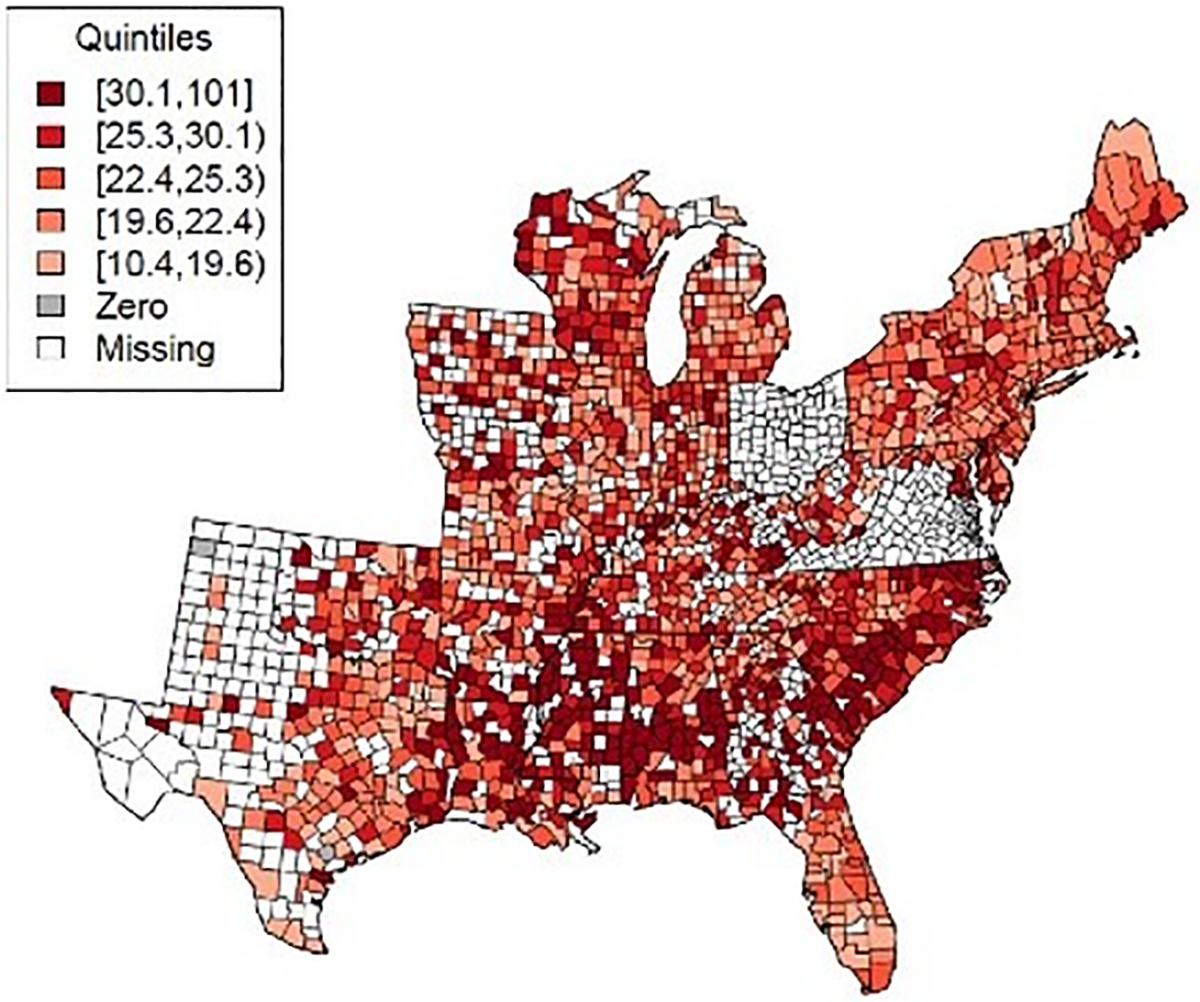
Prostate cancer mortality rates by county in the study region: 2006–2010. Note: 1. Counties labeled “missing” have incomplete prostate cancer mortality or incidence data. 2. Quintiles were calculated using only counties with non-zero values.

In the study sample, the mean county-level age-adjusted prostate cancer incidence rate was 141.20 cases per 100,000 men and the mean county-level age-adjusted prostate cancer mortality rate was 25.55 per 100,000 men (S1 Table). These rates should not be interpreted as national or regional averages because counties with too few deaths or cases were excluded prior to calculation. The mortality rates showed positive spatial autocorrelation (Moran’s I = 0.30, p-value < 0.001), indicating significant clustering. The counties with the highest observed prostate cancer mortality rates were located in the South (Fig 1). The urologist density distribution was right-skewed, with a median of 1.33 and mean of 2.10 urologists per 100,000 people. The reasons for this skew include many counties sharing the minimum value of zero and some counties having abnormally high values (S4 Fig). About 43% of those counties with complete data had a urologist density of zero, with most of them located outside the Northeast. 40% of counties had at least one radiation oncologist. The primary care physician density was 56.67 per 100,000 people. 46% of counties were classified as metropolitan area.

### Linear regression

In multivariate linear regression, we found an overall negative association between prostate cancer mortality and increasing urologist density (Table 1). Increasing urologist density by 1 urologist per 100,000 people, holding other variables constant, resulted in an expected decrease in prostate cancer mortality of -0.499 deaths per 100,000 men (95% CI -0.709 to -0.289, p-value < 0.001), or a 1.5% decrease. The presence of an additional radiation oncologist was not associated with a change in mortality. Increased incidence was associated with increased prostate cancer mortality, as was a higher level of non-white county population and classification as a HPSA. Increased levels of median income, higher levels of education in the county, and metropolitan status were associated with decreased prostate cancer mortality. Of note, the number of hospital beds, and percentage of the population over 65 years of age were not associated with a change in prostate cancer mortality.

**Table 1:** Coefficient Estimates in the Multivariate Ordinary Least Squares Regression of County Level Prostate Cancer Mortality Rates (per 100,000 men).

	Coefficient Estimate	95% CI	p-value
Prostate cancer incidence rate per 100,000 men	0.021	(0.007, 0.035)	**0.004**
Urologists per 100,000 people	-0.499	(-0.709, -0.289)	**< 0.001**
Radiation oncologists per 100,000 people	-0.169	(-0.584, 0.245)	0.423
Primary care MDs per 100,000 people	0.025	(0.005, 0.045)	**0.015**
Primary Care: If county is HPSA	0.802	(0.001, 1.603)	**0.050**
Hospital beds per 100,000 people (hundreds)	-0.070	(-0.230, 0.089)	0.389
Metropolitan county, binary	-1.821	(-2.737, -0.905)	**< 0.001**
Percent of population over 65 years old	-0.016	(-0.136, 0.104)	0.795
Per capita income, $1000s	-0.081	(-0.152, -0.011)	**0.024**
Percent of population non-white	0.223	(0.194, 0.252)	**< 0.001**
Percent of population over 25 with high school diploma	-0.137	(-0.213, -0.061)	**< 0.001**

Note: RSS = 78237.83; AICc = 10168.14; Adjusted R^2^ = 0.2889

95% confidence intervals calculated assuming normally distributed errors: Estimate ± 1.96 × standard error.

### Geographically weighted regression

Table 2 summarizes the estimated regression coefficients from each county from the GWR model. Goodness-of-fit statistics indicated that the GWR model better represented the data than the OLS model. The direction of associations in GWR were largely the same as in the OLS model based on a comparison of the mean and median coefficient estimates in the GWR model and the coefficient estimates in the OLS model.

**Table 2:** Range of Coefficient Estimates in the Multivariate Geographically Weighted Regression of County Level Prostate Cancer Mortality Rates (per 100,000 men).

	Min.	1st Qu.	Median	Mean	3rd Qu.	Max.
Intercept	-3.369	-1.012	-0.213	-0.420	0.265	
Prostate cancer incidence rate per 100,000 men	-0.035	0.003	0.013	0.014	0.022	0.057
Urologists per 100,000 people	-0.916	-0.550	-0.476	-0.468	-0.388	0.034
Radiation oncologists per 100,000 people	-0.892	-0.117	-0.003	0.000	0.131	0.685
Primary care MDs per 100,000 people	-0.053	0.006	0.013	0.012	0.021	0.049
Primary Care: If county is HPSA	-1.405	0.271	0.629	0.782	1.269	2.449
Hospital beds per 100,000 people (hundreds)	-0.331	-0.178	-0.098	-0.074	0.025	0.304
Metropolitan county, binary	-3.922	-1.810	-1.204	-1.154	-0.459	2.027
Percent of population over 65 years old	-0.257	-0.099	-0.003	-0.003	0.082	0.503
Per capita income, $1000s	-0.548	-0.038	0.004	-0.002	0.067	0.153
Percent of population non-white	0.039	0.152	0.202	0.204	0.257	0.334
Percent of population over 25 with high school diploma	-0.546	-0.369	-0.264	-0.236	-0.114	0.353

Note: RSS = 56233.7; AICc = 10010.2; Adjusted R^2^ = 0.3892

Model fit statistics suggest that the GWR model fit our data better than the OLS model. Comparing the GWR model to the OLS model, RSS and AICc were lower, and the Adjusted R^2^ was higher with GWR.

Intercept estimates are non-zero because mean-centering was done globally, not locally.

**Fig 2:**
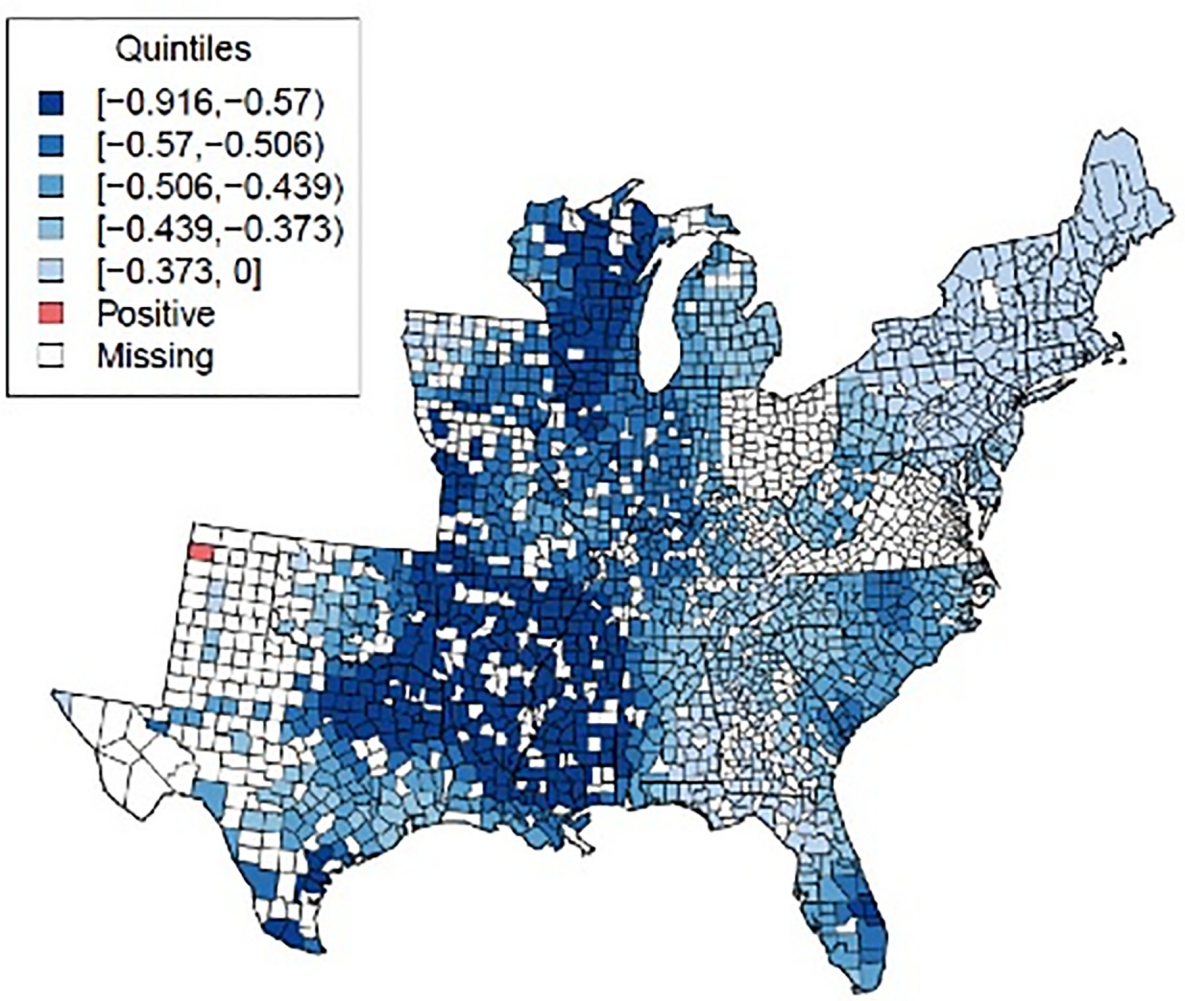
Expected change in prostate cancer mortalities using GWR model, given a one unit increase in urologist density, while holding other covariates constant (not considering local measures of significance). Note: 1. Counties part of the [-0.373,0.034] quintile with coefficient estimates greater than zero are shown in red. Counties of that same quintile with negative coefficient estimates are shown in the lightest blue.

Fig 2 maps the results from the GWR model, showing the predicted change in prostate cancer mortality for a one unit increase in urologist density per 100,000 people, holding all other covariates constant, without considering the local adjusted p-values. This depicts significant spatial variation in predicted change in prostate cancer mortalities. The region with the largest predicted decrease in prostate cancer mortality consists of counties in the southern Mississippi River states of Arkansas, Mississippi, and Louisiana, as well as parts of Illinois, Indiana, and Wisconsin. This means that in these counties, all else being equal, a one-unit increase in urologist density (one urologist per 100,000 men) is associated with a with lower prostate cancer mortality than in counties with coefficients closer to zero. Only one county out of 1,492 had an estimated urologist density coefficient that was greater than zero. Some counties had two or more coefficients with high (greater than 0.5) variance decomposition proportions (VDPs, S5 Fig), which suggests a problem with collinearity in the GWR model in those regions.

**Fig 3:**
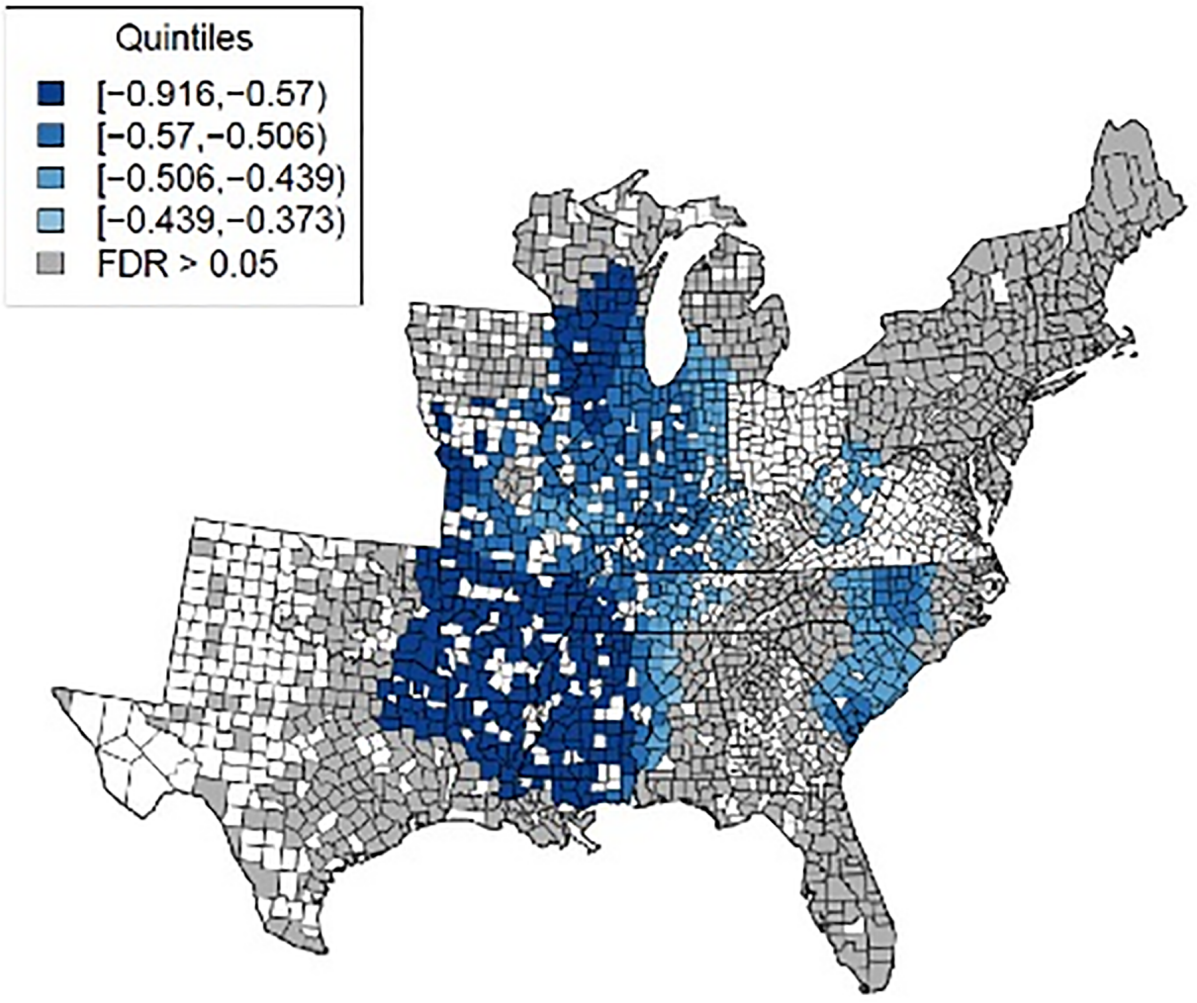
Expected change in prostate cancer mortalities using GWR model, given a one unit increase in urologist density, while holding other covariates constant (only showing counties with adjusted approximate p-value ≤ 0.05). Note: Quintiles refer to coefficient estimates from all counties regardless of significance level.

When limiting visualization of the predicted change in prostate cancer mortality to counties with adjusted approximate p-values below 0.05 (Fig 3), a smaller number of counties had statistically significant predicted decreases in mortality with increased urologist density. Therefore, there is higher confidence in a true negative relationship between urologist density and prostate cancer mortalities in these areas. The addition of one urologist per 100,000 people in these areas is associated with decrease of 0.411 to 0.916 in prostate cancer mortality per 100,000 men (1.6–3.6%).

### Limitations

Although GWR allows modeling of spatially varying regression effects, it should be used with caution. GWR is an exploratory method and does not provide exact statistical inference on regression relationships. Coefficient estimates of counties had zero urologists cannot be easily used to explain cancer mortality. The model can be unstable and sensitive to the set of covariates used as input, particularly when variables are correlated. Therefore, model selection and diagnosis is an especially important part of the analysis when using GWR. In addition, the spatial patterns of the parameter estimates can be due to model misspecification.[15] In this study, variable selection was based on diagnostics procedures and previous research in cancer mortality. Moreover, as an ecological analysis, our study did not contain covariate and outcome values for individuals, and therefore we cannot use our findings to make inferences about individual patients. While the data used in this study are maintained by Federal agencies and are of high quality, sampling error in the data collection designs may be an issue.

## Discussion

Overall, increased urologist density was associated with decreased prostate cancer mortality in the OLS model, which is consistent with prior findings. However, our exploratory spatial analysis revealed a complex relationship between prostate cancer mortality and urologist supply. The GWR analysis confirmed the results from the OLS model in terms of coefficient sign for much of the study region, but also demonstrated that the strength and confidence of the association between urologist density and prostate cancer mortality varies across the study region, with stronger negative effects found in southern Mississippi River region as well as parts of Illinois, Indiana, and Wisconsin. In addition to strong negative associations, these areas also had higher prostate cancer mortality rates and relatively low urologist density. As a result, these areas could be targeted for increasing the supply of urologists, as it associated with the largest predicted improvement in prostate cancer mortality.

The mechanisms underlying the spatially non-stationary association between urologist density and prostate cancer mortality cannot be addressed in this study, but we offer some potential explanations for this phenomenon. Urologist supply might have a diminishing marginal effect on prostate cancer mortalities. It is not rational to expect that a one unit increase in urologist supply in a high density region would have the same effect as in a low density region such as the southern Mississippi River region. Similar results were reported in prior work when urologist density was grouped into 4 categories, only showing a statistically significant change in prostate cancer mortality when increasing urologist density above zero, with no additional impact after that.[7] Individual patient factors, such as the disease characteristics (e.g., tumor stage, tumor size, receptor status, and comorbidity) and socioeconomic status (e.g., insurance coverage, household income, education level, and race/ethnicity), may also be a factor in the non-stationary relationship between urologist density and prostate cancer mortality. This may lead to future research using multilevel modeling to incorporate both individual patient and contextual variables in analysis. However, it is difficult to acquire individual-level data for non-SEER regions.

Prior work attempting to associate cancer specific mortality with physician density uses linear or logistic regression, which do not account for spatial non-stationary effects.[7,32,33] We adopted a geographically weighted regression approach to supplement a global regression model in order to examine spatial non-stationarity in the relationship between physician supply and cancer mortalities. We replicated findings of the earlier studies using different data suggesting negative association between specialist supply and cancer mortalities and in addition we contribute new substantive insights by investigating the role of place. The improved performance of GWR, which provides a local model of the variables by fitting a regression equation to every observation in the dataset, over the OLS regression model was demonstrated by model fit measures. GWR provides cancer care researchers an exploratory tool supplementary to the OLS regression model to investigate how relationships between variables vary across the study region.

This study has several implications for cancer care research. First, the non-stationary association between urologist density and prostate cancer suggests regional variation of an ecological relationship. As the final GWR coefficient map (Fig 3) suggests, the effect of urology supply may be more important in certain areas than others in the United States, which calls for place-specific or place-sensitive forms of analysis.[34,35] This study also sheds light on where to focus and modify cancer care policies by revealing non-stationary associations. Explicitly, our findings offer an empirical basis for locally tailored policy formation, which may improve the efficiency of cancer care.

Future research seeking to examine the potential impact of physician supply on quality of cancer care would benefit from incorporating spatial heterogeneity with regard to cancer care dynamics. Further work is also needed to understand the effect of physician supply on cancer care at the individual patient level. Finally, longitudinal data on all cause mortalities, healthcare resources, and socio-demographic factors at the county level can improve predictive ability. Current cancer mortality/incidence data is too scarce at county-level to perform spatial panel data analysis.

### Conclusions

Increasing urologist density was associated with decreased prostate cancer mortality rates and the strength of that association varied across the study region, with larger effects and greater confidence in the southern Mississippi River states of Arkansas, Mississippi, and Louisiana, as well as parts of Illinois, Indiana, and Wisconsin.

## Supporting Information

S1 Fig Residuals from the OLS model. (PDF)

S2 Fig Local Indicators of Spatial Association of residuals from the OLS Model. (PDF)

S3 Fig Counties with complete prostate cancer incidence/mortality data. (PDF)

S4 Fig Urologist density by county in the study region: 2006–2010. (PDF)

S5 Fig GWR collinearity diagnostics. (PDF)

S1 File GWR and FDR methods. (PDF)

S1 Table Descriptive Statistics of Outcome and Explanatory Variables (n = 1492 counties). (PDF)
