# Supplementary material for: Geographic Analysis of Urologist Density and Prostate Cancer Mortality in the United States
Source: PLoS One. 2015 Jun 25;10(6):e0131578. doi: 10.1371/journal.pone.0131578 (PMC4482500; doi:10.1371/journal.pone.0131578)
Supplement: S1 Fig — (PDF) [file pone.0131578.s001.pdf]

S1 Fig. Residuals from the OLS model

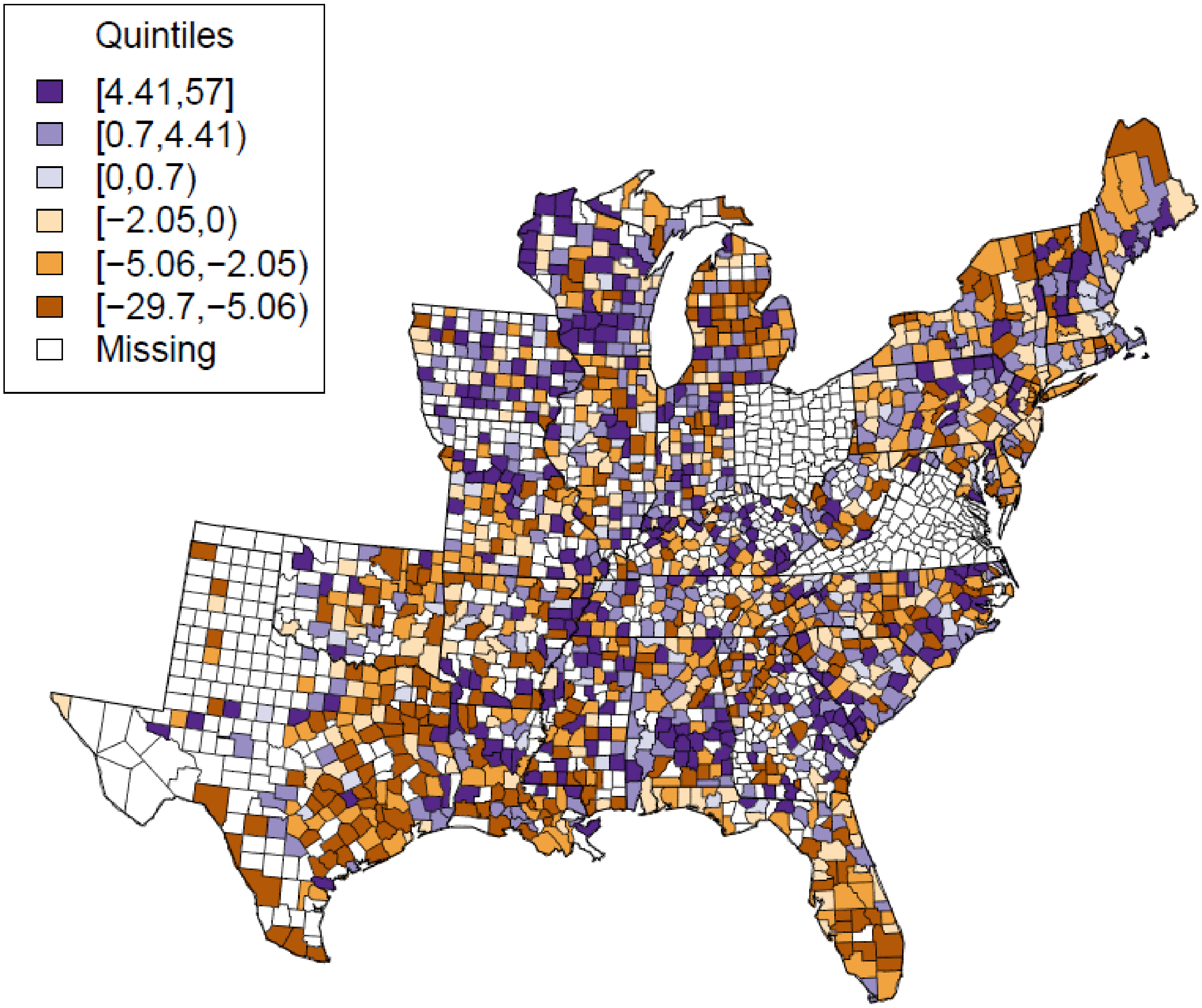

Note:

1. Independent variables in the model include urologists per 100K, radiation oncologists per 100K, primary care MDs per 100K, incidence per 100K men, hospital beds per 100K, HPSA, metropolitan status, the percentages of population over 65, non-white, and over 25 without a high school diploma, and per capita income.
2. Moran's I=0.121 ($p<0.001$)
3. We calculated quintiles but then split the middle group in two because it spanned zero. The original middle quintile was [-2.05,0.7).
