# Supplementary material for: Geographic Analysis of Urologist Density and Prostate Cancer Mortality in the United States
Source: PLoS One. 2015 Jun 25;10(6):e0131578. doi: 10.1371/journal.pone.0131578 (PMC4482500; doi:10.1371/journal.pone.0131578)
Supplement: S2 Fig — (PDF) [file pone.0131578.s002.pdf]

S2 Fig. Local Indicators of Spatial Association of residuals from the OLS Model

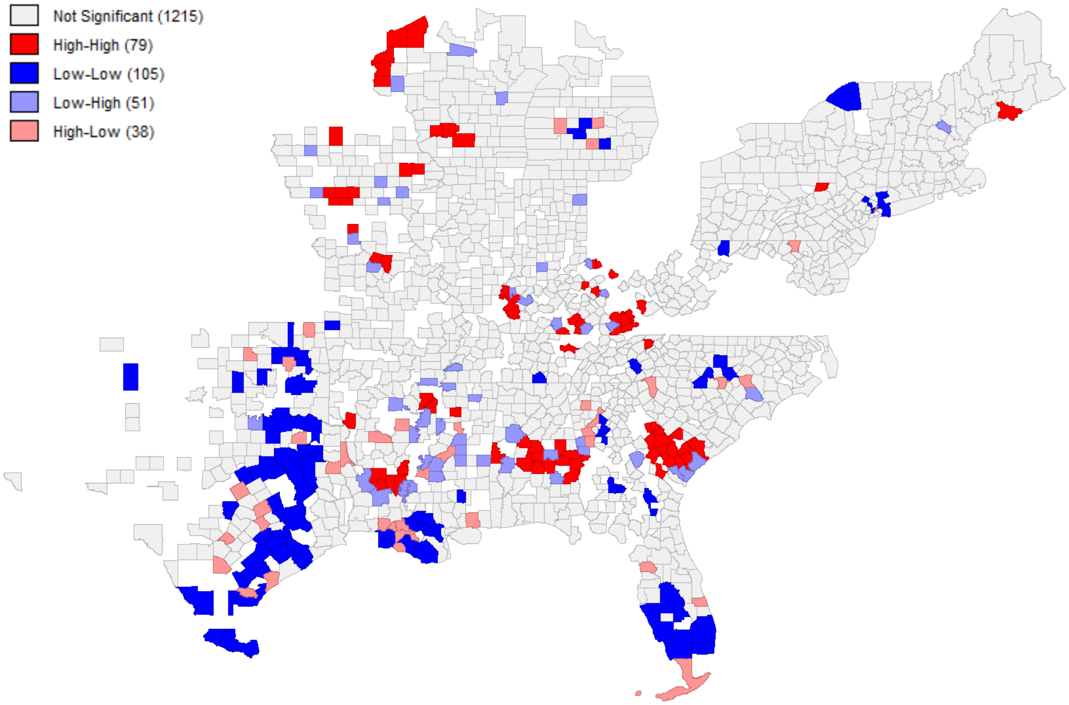

Note:
1. The high-high and low-low locations (positive local spatial autocorrelation) are typically referred to as spatial clusters
2. The high-low and low-high locations (negative local spatial autocorrelation) are termed spatial outliers.
3. Spatial clusters shown on the cluster map only refer to the core of the cluster.
