# Supplementary figures and images for: Geographic Analysis of Urologist Density and Prostate Cancer Mortality in the United States
Source: PLoS One. 2015 Jun 25;10(6):e0131578. doi: 10.1371/journal.pone.0131578 (PMC4482500; doi:10.1371/journal.pone.0131578)

S3 Fig. Counties with complete prostate cancer incidence/mortality data

■ Have data

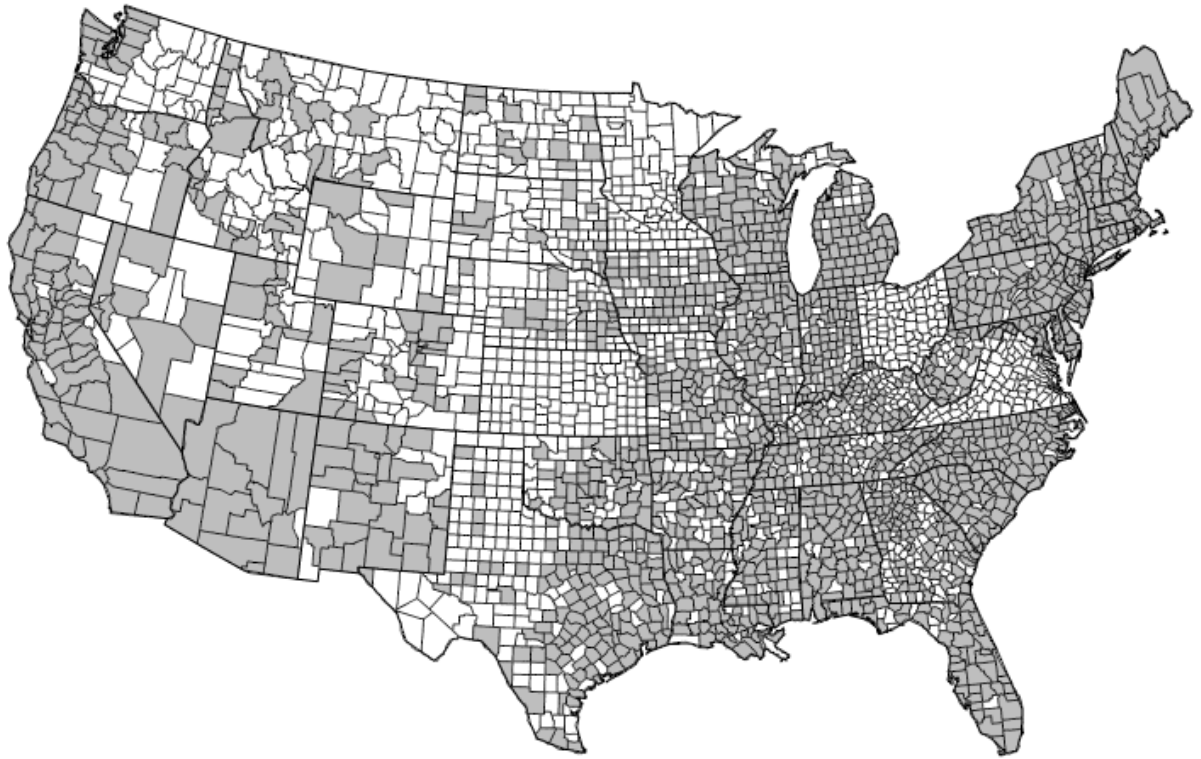

Supplement: S3 Fig — (PDF) [file pone.0131578.s003.pdf]
