# Supplementary material for: Geographic Analysis of Urologist Density and Prostate Cancer Mortality in the United States
Source: PLoS One. 2015 Jun 25;10(6):e0131578. doi: 10.1371/journal.pone.0131578 (PMC4482500; doi:10.1371/journal.pone.0131578)
Supplement: S4 Fig — (PDF) [file pone.0131578.s004.pdf]

S4 Fig. Urologist density by county in the study region: 2006-2010

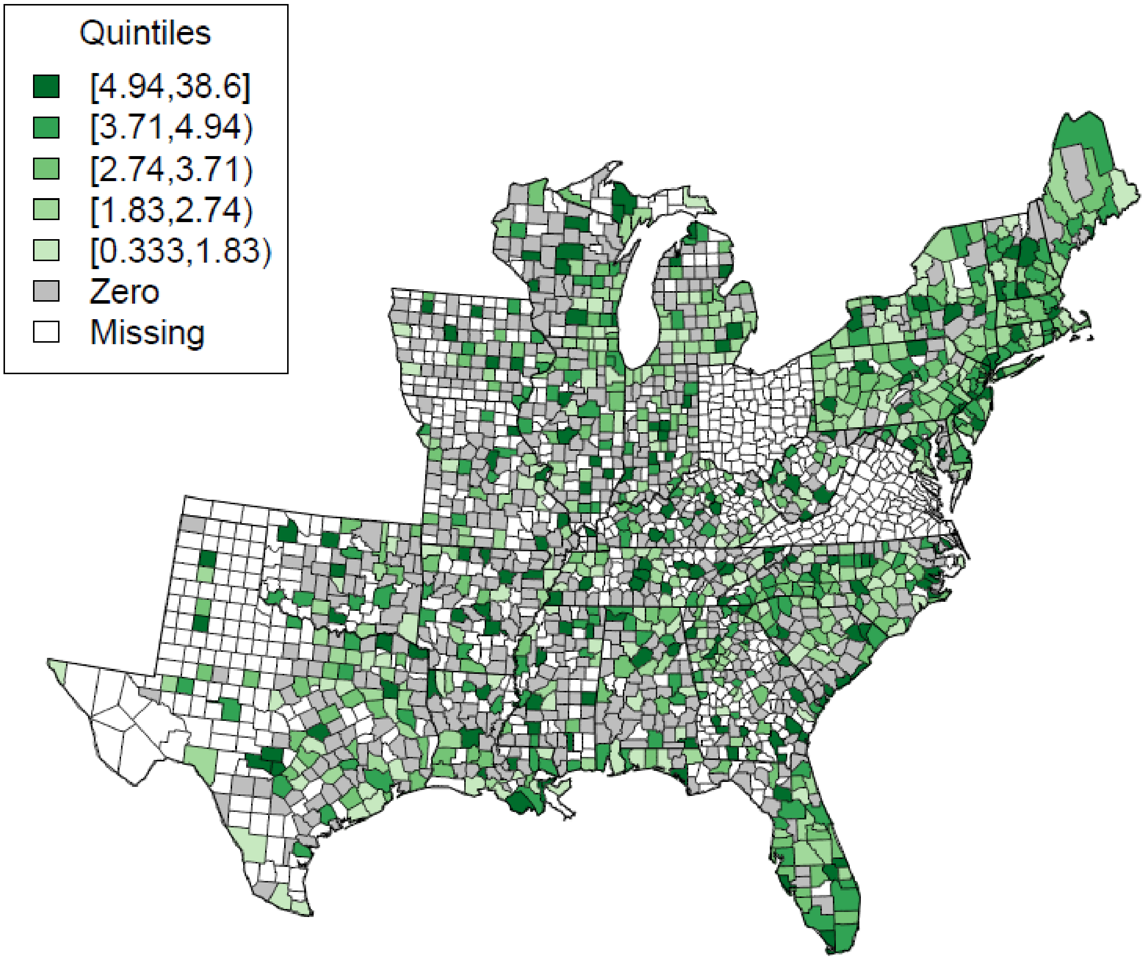

Note:

1. Counties labeled “missing” have incomplete prostate cancer mortality or incidence data.
2. 47.0% of eastern counties with complete data had a urologist density of zero.
3. Quintiles were calculated using only data from counties with non-zero urologist density.
