# Supplementary material for: Geographic Analysis of Urologist Density and Prostate Cancer Mortality in the United States
Source: PLoS One. 2015 Jun 25;10(6):e0131578. doi: 10.1371/journal.pone.0131578 (PMC4482500; doi:10.1371/journal.pone.0131578)
Supplement: S5 Fig — (PDF) [file pone.0131578.s005.pdf]

S5 Figure. GWR collinearity diagnostics

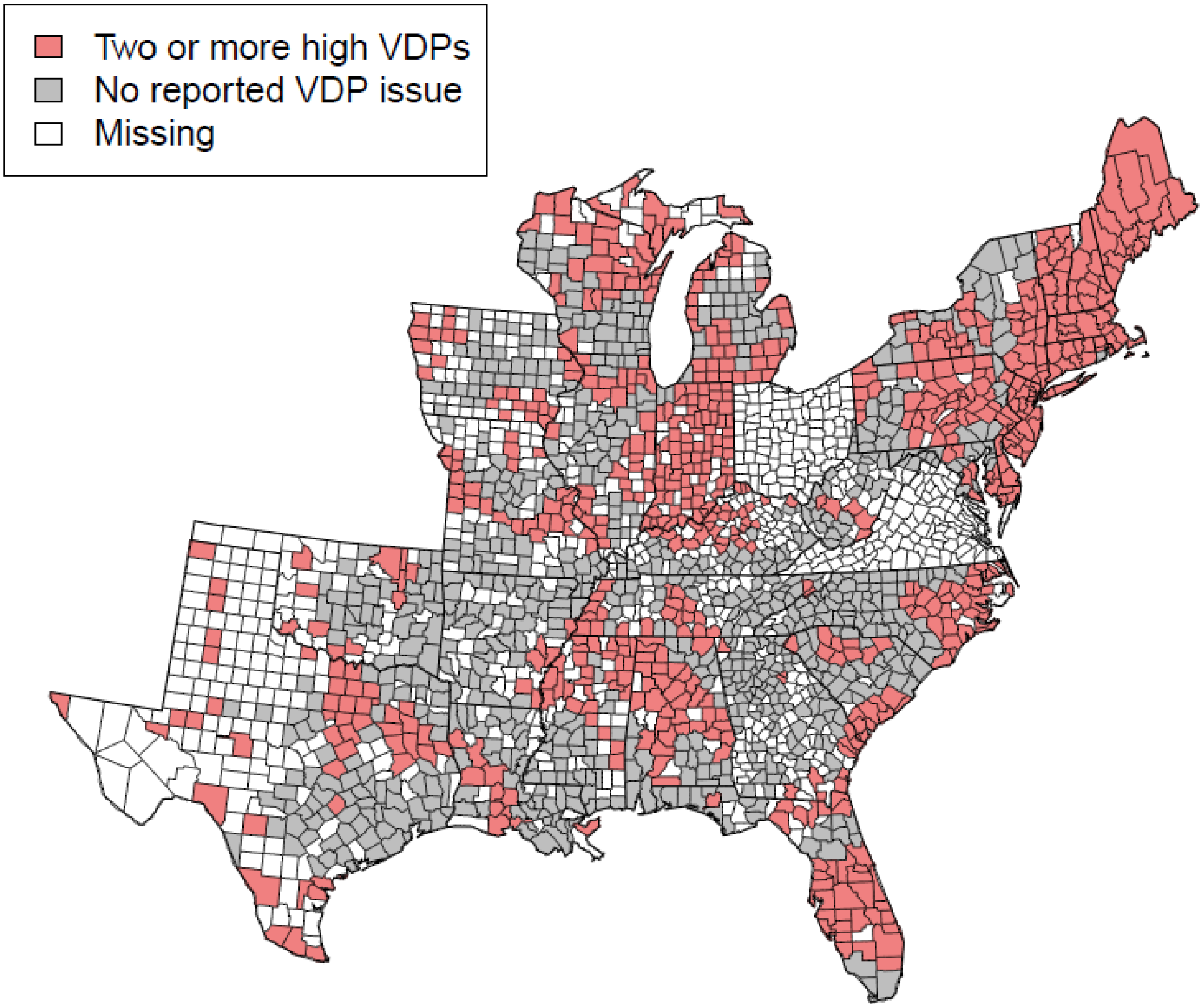

Note:
1. Counties in red were flagged as having two or more variance decomposition proportions (VDPs) exceeding 0.5 (model not including insurance rates).
2. Only El Paso County, Texas had a condition index above 30 in addition to two high VDPs, indicating a more serious collinearity issue.
