# Supplementary material for: Geographic Analysis of Urologist Density and Prostate Cancer Mortality in the United States
Source: PLoS One. 2015 Jun 25;10(6):e0131578. doi: 10.1371/journal.pone.0131578 (PMC4482500; doi:10.1371/journal.pone.0131578)
Supplement: S1 File — (PDF) [file pone.0131578.s006.pdf]

S1 File. GWR and FDR methods

Geographically weighted regression (DW) assumes the relationships between terms in a regression model are not constant across space. In GWR, there is an underlying spatial distribution of data points (in our study, the county centroids). The estimated effect size may be larger in one area than another, and treating all sites the same by ignoring their geographic relationship could oversimplify results or give misleading conclusions. Thus, the results of GWR includes local coefficient estimates that can be mapped to reveal possible regional trends.[1, 2]

GWR involves model specification much like that of simple linear regression. The main differences are that GWR uses a spatial weights matrix, and that in GWR one must calculate regression coefficients at every data point. In simple linear regression, the outcome (*y*) is modeled for every data point as

$$y_i = \beta_0 + \sum_{k=1}^{m} \beta_k x_{ik} + \varepsilon_i$$

where β can be estimated by $\widehat{\boldsymbol{\beta}} = (\boldsymbol{X}^T\boldsymbol{X})^{-1}\boldsymbol{X}^T\boldsymbol{y}$.

In GWR, the estimated coefficients $\widehat{\boldsymbol{\beta}}$ differ from site to site. $\widehat{\boldsymbol{\beta}}$ gets estimated at every site but with places farther away having less influence on the outcome based on a spatial weights matrix $\boldsymbol{W_i}$. The diagonal of the spatial weights matrix controls how much influence each site has according to a function decreasing in the distance between sites. Off-diagonal values are zero. Thus, using GWR one estimates a site-specific $\widehat{\boldsymbol{\beta}_i} = (\boldsymbol{X}^T\boldsymbol{W}_i\boldsymbol{X})^{-1}\boldsymbol{X}^T\boldsymbol{y}$. [1, 2]

We performed our analyses in R (Version 3.0.2) using packages GWmodel and gwrr. With the GWmodel function bw.gwr, we used a cross-validation approach to select a bandwidth assuming an exponential kernel. Cross-validation is a statistical method for estimating a parameter. The kernel defines the weight assigned to a site *j* when some site *i* is being analyzed, and the bandwidth is a parameter of the kernel. We used an exponential kernel such that, for a given bandwidth (*b*), the weight (*w(i,j)*) for points separated by some distance (*d(i,j)*) is

$$w(i,j) = exp\left(-\frac{d(i,j)}{b}\right),$$

. With the kernel function defined and the bandwidth estimated, we used the function gwr.basic to compute local coefficient estimation for each site in our data set.

We approximated the significance of local coefficient estimates using the GWmodel function gwr.t.adjust. We found adjusted approximate p-values based on the Benjamani-Yekutieli false discovery rate (FDR) method, which accounts for multiple testing and positive dependency.[3, 4] The p-values from t-tests of regression coefficients in GWR are only approximate due to reusing the data to estimate the kernel bandwidth and the regression coefficients.[5] The FDR is the expected probability of falsely rejecting a null hypothesis.

Collinearity diagnostics

Collinearity issues have been found to be especially problematic in GWR models.[5] We used diagnostic tools from the R package gwrr to explore problems with collinearity in the GWR models.[6] To identify problems with local collinearity, we examined variance-decomposition proportions (VDPs) and condition indexes for every local GWR model using the gwr.vdp function. When a large (above 30) condition index for a given site is paired with two or more variables with a high (> 0.5) VDPs, there may be an issue with local collinearity. Consequences of local collinearity include estimated regression coefficients with increased magnitude and counterintuitive signs, inflated variances of regression coefficients, and insignificant statistical test values.[5] In our final GWR model, only El Paso County, Texas, on the western tip of the state, had a large (above 30) condition index paired with two variables with a high (> 0.5) VDPs (Appendix Figure 4).

Diagnostic tools such as gwr.vdp are important to use in the model selection process by identifying variables exhibiting locally collinear behavior. For example, we initially considered the variable for percentage of the population under 65 without health insurance as a covariate. The diagnostic tools revealed that with the health insurance variable in the model, 59% of the counties (n=1492) had two or more variables with high VDPs, while only 48% of counties had the same problem when we excluded that variable from the model (Appendix Figure 4). Thus, the diagnostic software identified a variable that should be removed to reduce the negative effects of collinearity.

References

1. Fotheringham AS, Brunsdon C, Charlton M: Geographically Weighted Regression: The Analysis of Spatially Varying Relationships. Wiley, 2002

2. Brunsdon C, Fotheringham AS, Charlton ME: Geographically Weighted Regression: A Method for Exploring Spatial Nonstationarity. Geogr Anal 28:281–298, 1996

3. Benjamini Y, Yekutieli D: The Control of the False Discovery Rate in Multiple Testing under Dependency. Ann Stat 29:1165–1188, 2001

4. Byrne G, Charlton M, Fotheringham S: Multiple dependent hypothesis tests in geographically weighted regression, in 10th International conference on geocomputation. UNSW, Sydney November–December. 2009

5. Wheeler DC: Diagnostic tools and a remedial method for collinearity in geographically weighted regression. Environ Plan A 39:2464 – 2481, 2007

6. David Wheeler: R Package “gwrr” [Internet][cited 2013 Dec 4] Available from: http://cran.r-project.org/web/packages/gwrr/gwrr.pdf
