# Supplementary material for: Geographic Analysis of Urologist Density and Prostate Cancer Mortality in the United States
Source: PLoS One. 2015 Jun 25;10(6):e0131578. doi: 10.1371/journal.pone.0131578 (PMC4482500; doi:10.1371/journal.pone.0131578)
Supplement: S1 Table — (PDF) [file pone.0131578.s007.pdf]

S1 Table. Descriptive Statistics of Outcome and Explanatory Variables (*n* = 1492 counties)

	**Median**	**Interquartile range**	**Moran's I**
Prostate cancer mortality rate per 100,000 men	23.80	(20.20 – 28.60)	0.3037***
Prostate cancer incidence rate per 100,000 men	140.00	(121.00 – 159.50)	
Urologists per 100,000 people	1.33	(0.00 – 3.50)	
Radiation oncologists per 100,000 people	0.00	(0.00 – 1.22)	
Primary care MDs, per 100,000 people	52.16	(37.09 – 70.30)	
Proportion of counties classified as HPSA	0.34	–	
Hospital beds per 100,000 people, (hundreds)	2.39	(1.30 – 3.89)	
Metropolitan county, binary	0.46	–	
Percent of population over 65 years old	14.89	(12.94 – 16.97)	
Per capita income, $1000s	31.86	(28.69 – 36.13)	
Percent of population non-white	12.80	(5.50 – 27.95)	
Percent of population over 25 with high school diploma	83.95	(78.40 – 87.90)	

Notes:
1. The GeoDA software package and a weight matrix of 6 nearest neighbors was used to compute Moran's I.
2. *** Moran's I is significant at $p<0.001$ in the permutation tests
